# Cross-Sectional Study of Clinical Predictors of Coccidioidomycosis, Arizona, USA 

**DOI:** 10.3201/eid2806.212311

**Published:** 2022-06

**Authors:** Ferris A. Ramadan, Katherine D. Ellingson, Robert A. Canales, Edward J. Bedrick, John N. Galgiani, Fariba M. Donovan

**Affiliations:** University of Arizona, Tucson, Arizona, USA (F.A. Ramadan, K.D. Ellingson, E.J. Bedrick);; George Washington University, Washington, DC, USA (R.A. Canales);; University of Arizona College of Medicine–Tucson, Tucson (J.N. Galgiani, F.M. Donovan)

**Keywords:** coccidioidomycosis, Coccidioides, fungi, respiratory infections, Valley fever, risk factors, prediction model, diagnosis, Arizona, United States

## Abstract

Demographic and clinical indicators have been described to support identification of coccidioidomycosis; however, the interplay of these conditions has not been explored in a clinical setting. In 2019, we enrolled 392 participants in a cross-sectional study for suspected coccidioidomycosis in emergency departments and inpatient units in *Coccidioides*-endemic regions. We aimed to develop a predictive model among participants with suspected coccidioidomycosis. We applied a least absolute shrinkage and selection operator to specific coccidioidomycosis predictors and developed univariable and multivariable logistic regression models. Univariable models identified elevated eosinophil count as a statistically significant predictive feature of coccidioidomycosis in both inpatient and outpatient settings. Our multivariable outpatient model also identified rash (adjusted odds ratio 9.74 [95% CI 1.03–92.24]; p = 0.047) as a predictor. Our results suggest preliminary support for developing a coccidioidomycosis prediction model for use in clinical settings.

Coccidioidomycosis, colloquially known as cocci or Valley fever, is a fungal infection endemic to the southwestern United States and parts of Central and South America (*1*). Infection occurs through inhalation of an arthroconidium from the dimorphic, soil-dwelling fungi *Coccidioides immitis* and *C. posadasii*. Incidence has increased since 1995, when coccidioidomycosis became a reportable infection (*2*). During 2016–2018, the Centers for Disease Control and Prevention reported a 32% increase in coccidioidomycosis cases (*3*). Epidemiologic studies suggest climate change, more frequent soilborne dust exposures, and a growing population of older adults in endemic regions as possible causes for increased coccidioidomycosis rates (*4*). Despite enhanced surveillance efforts, coccidioidomycosis incidence is underreported (*4*,*5*), and estimates suggest ≥150,000 infections annually in the United States (*6*).

Because of limited ability to prevent *Coccidioides* exposure in the community and no existing vaccine, coccidioidomycosis poses a substantial burden to patients and healthcare systems in endemic areas (*7*,*8*). Most (60%) *Coccidioides* infections are subclinical, but clinical cases produce protracted respiratory conditions (*9*,*10*). Observational studies indicate that 15%–29% of community-acquired pneumonia in endemic areas is caused by coccidioidomycosis (*11*,*12*). Diverse and nonspecific manifestations including fatigue, cough, fever, and rash make diagnosis challenging, and coccidioidomycosis can easily be mistaken for other respiratory illnesses, eczema, or bacterial pneumonia. Thus, misdiagnosis and inappropriate treatments are common, and <81% of patients are prescribed an antibacterial drug (*5*,*12*). However, few studies have investigated factors associated with increased coccidioidomycosis incidence to support clinical decision-making (*13*).

Increased incidence and complex clinical manifestations of coccidioidomycosis emphasize the need to improve disease identification in clinical settings. In 2019, we prospectively enrolled participants with suspected coccidioidomycosis to evaluate a novel diagnostic test (*14*). For this study, we used data from our prior study to develop a coccidioidomycosis prediction model based on demographic, clinical, and laboratory factors. We developed independent models for outpatient and inpatient settings.

## Methods

### Design

During January–December 2019, we collected data from a prospective study that enrolled participants at 2 academic medical centers in southern Arizona, Banner-University Medical Center Tucson, and Banner-University Medical Center Phoenix, and their affiliated outpatient clinics. During that study, we enrolled 402 participants with suspected coccidioidomycosis, which was defined by clinician orders for coccidioidomycosis serologic testing (*14*). Our protocol was consistent with public health recommendations to test for coccidioidomycosis among patients with pneumonia-like symptoms in endemic areas. Patients with alternative clinical manifestations, such as fibrocavitary or disseminated disease, were also evaluated for coccidioidomycosis. Research coordinators were alerted to potential participants via electronic medical record (Cerner, https://www.cerner.com), when clinicians ordered a coccidioidomycosis screening test, or directly by outpatient clinicians (*15*). We excluded persons <18 years of age or with a history of coccidioidomycosis. Consenting participants completed a medical questionnaire and provided an additional blood sample (*14*). The University of Arizona Institutional Review Board provided research approval to enroll participants (project no. 1811085933A011).

### Variables

Coccidioidomycosis was our primary outcome of interest, which we defined as confirmatory evidence via positive *Coccidioides* serologic testing, such as ELISA, immunodiffusion, compliment fixation titers >1:2, or a positive culture. We coded indeterminate ELISA and immunodiffusion results as negative. Demographic data collected included age, sex, race, ethnicity, and length of residence in an endemic area. Participants or their designated proxies reported previous symptoms and length of illness via survey. Laboratory measurements were leukocyte count and differential, hemoglobin, platelet count, serum albumin, and total serum protein. Participants provided an additional blood sample that was used to measure C-reactive protein (CRP), erythrocyte sedimentation rate (ESR), and procalcitonin (PCT) levels. A team of physicians conducted a review of each participant’s chart to compile any history of immunocompromised status, such as type 2 diabetes, HIV/AIDS, or immunosuppressive therapies. We identified coccidioidomycosis clinical manifestations by using diagnostic notes and radiographic results.

### Analysis

We stratified our analyses by inpatient versus outpatient admission status because of systematic differences in the complexity of clinical presentation and availability of electronic medical record data. We classified race as a binary White or non-White variable because of the low representation of minority racial groups. Continuous variables displayed non-normal distributions. We used the nonparametric Mann-Whitney U test to evaluate the distribution of continuous variables across groups and Fisher exact test to evaluate categorical variables across groups.

Before model development, we evaluated potential predictor variables for multicollinearity by using variance inflation factors and correlation. We applied a correlation threshold of *r*>0.7 and identified eosinophil percentage as a colinear feature. We omitted eosinophil percentage from our analysis because we considered it to be less clinically relevant in contrast to eosinophil count (Appendix Figures 1, 2). All numeric variables exhibited nonnormal distributions and were log transformed. We included clinical features, participant symptoms, and age as binary variables within models, and incorporated length of residence, duration of illness, and laboratory markers as continuous measures. To reduce the loss of sample size, we imputed missing data by Gibbs sampling (*16*,*17*). We evaluated imputed data stability by replicating variable selection methods for 5 distinct completed datasets. In brief, we imputed numeric variables by using predictive mean matching, we imputed binary variables with logistic regression, and we imputed multiclass variables by using Bayesian polytomous regression. An average of 12 (3.1%) observations were missing from each variable; however, <63 (16.1%) observations were missing for any single feature. Data with the highest number of missing observations were eosinophil count (16.1%), albumin (13.6%), and total protein (13.6%) (Appendix Table 1). We used imputation methods to retain a sufficient sample size for feature selection; listwise deletion resulted in a loss of 156 (40%) observations. 

### Variable Selection and Evaluation

First, we developed univariable logistic regression models, reporting all parameter estimates in terms of odds ratios (ORs) on imputed data. We constructed multivariable models by using the semi-automated least absolute shrinkage and selection operator (LASSO) method on imputed data (*18*). In brief, LASSO is a selection technique that uses penalization to shrink small regression coefficients to zero. Penalization (lambda) parameters can be selected by using a minimum cross-validated mean squared error (CVMSE) or the CVMSE <1 SD of the minimum. We used the mean of these 2 lambda values to penalize our models. We retained variables with nonzero coefficients in each model. We selected LASSO because of its ability to select influential features in a high-dimensional dataset (i.e., a high number of variables relative to the dataset). Other regression methods often suffer degeneracies when the number of predictors exceeds or is close to the number of observations (*19*).

We performed leave-one-out cross-validation to calculate predictive performance of multivariable models and obtain corrected estimates of sensitivity, specificity, and predictive values. This internal validation method provides an out-of-sample performance estimate of each model. We used receiver operating characteristic (ROC) area under the curve (AUC) to evaluate predictive performance of our models. ROC AUC uses a combination of sensitivity and specificity to assess predictive performance. An ROC AUC of 1.0 corresponds to perfect discrimination, whereas 0.50 indicates no predictive ability. We performed sensitivity analyses by using standardized laboratory reference ranges (*20*–*22*) and among participants with and without identifiable immunocompromised conditions. We developed supplemental models to identify alternative laboratory thresholds predictive of coccidioidomycosis. We used R version 3.6.3 (*23*) to conduct analyses and performed multiple imputation by using the mice package (*17*). We conducted LASSO by using the glmnet package in R (*24*). We considered p<0.05 statistically significant with no correction for multiple testing. We report this study according to STrengthening the Reporting of OBservational studies in Epidemiology (STROBE) guidelines (https://www.strobe-statement.org) (Appendix Tables 1–9).

## Results

Median participant age was 57 years; 48.8% of participants were female and 51.2% male (Table 1). Participants self-reported as White (82.9%), African American (6.8%), American Indian/Alaskan Native (4.5%), and Asian (1.8%). Only 18.6% of participants tested positive for coccidioidomycosis. The median age for coccidioidomycosis-positive participants was 55 years, and median age for coccidioidomycosis-negative participants was moderately older at 57 years (p = 0.04). Coccidioidomycosis-positive participants had a shorter median length of residence, 13 years, than the 21 years for coccidioidomycosis-negative participants (p = 0.02). A higher proportion of non-White participants had a coccidioidomycosis-positive diagnosis (p = 0.02), but we did not identify statistically significant variations by sex (p = 0.51) or ethnicity (p = 0.88). Coccidioidomycosis-positive participants had significantly lower rates of immunocompromised conditions (33.3%) than did coccidioidomycosis-negative participants (55.1%) (p = 0.001). Positive participants had lower rates for symptoms including fatigue (p = 0.03) and shortness of breath (p = 0.02) than did negative participants, but positive participants had higher rates of rash (36.9%) than did negative participants (12.9%; p<0.001) (Appendix Table 2). Laboratory markers including PCT, CRP, and ESR were significantly lower in coccidioidomycosis-positive than -negative participants (p<0.001). Inversely, hemoglobin (p = 0.008), platelet count (p = 0.01), eosinophil count (p<0.001), and total protein (p = 0.04) levels were higher among coccidioidomycosis-positive participants (Appendix Table 2).

**Table 1:** Patient characteristics by confirmed *Coccidioides* diagnosis in a cross-sectional study of clinical predictors of coccidioidomycosis, Arizona, USA*

Characteristics	Positive, n = 73	Negative, n = 319	Total, n = 392	p value
Median age, y (range)	55 (18–83)	57 (18–98)	57 (18–98)	**0.038**
Sex, no. (%)				0.514
F	38 (52.8)	152 (47.9)	190 (48.8)	
M	34 (47.2)	165 (52.1)	199 (51.2)	
Race, no. (%)				**0.024**
African American	8 (11.4)	18 (5.8)	26 (6.8)	
American Indian/Alaska Native	6 (8.6)	11 (3.5)	17 (4.5)	
Asian	3 (4.3)	4 (1.3)	7 (1.8)	
White	52 (74.3)	263 (84.8)	315 (82.9)	
Unknown	1 (1.4)	14 (4.5)	15 (3.9)	
Ethnicity, no. (%)				0.882
Hispanic	18 (26.5)	87 (28.1)	105 (27.8)	
Non-Hispanic	50 (73.5)	223 (71.9)	273 (72.2)	
Median length of endemic residence, y (range)	13 (0–78)	21 (0–98)	20 (0–98)	**0.017**
Admission status, no. (%)				
Outpatient	31 (42.5)	49 (15.4)	80 (20.5)	**<0.001**
Inpatient	42 (57.5)	269 (84.6)	311 (79.5)	
Immunocompromised, no. (%)				**0.001**
Y	24 (33.3)	174 (55.1)	198 (51)	
N	48 (66.7)	142 (44.9)	190 (49)	


*Bold text indicates statistical significance.

Our initial sample consisted of 392 participants with suspected coccidioidomycosis (Figure). Participants were stratified into outpatient (n = 99) and inpatient groups (n = 293). The outpatient group consisted of 35 coccidioidomycosis-positive participants and 64 coccidioidomycosis-negative participants; our inpatient group consisted of 38 coccidioidomycosis-positive participants and 255 coccidioidomycosis-negative participants (Table 2). The median age for outpatients was 57 years for coccidioidomycosis-positive and 51 years for coccidioidomycosis-negative participants, but we noted no statistical difference in age (p = 0.53) (Table 2). 

**Figure:**
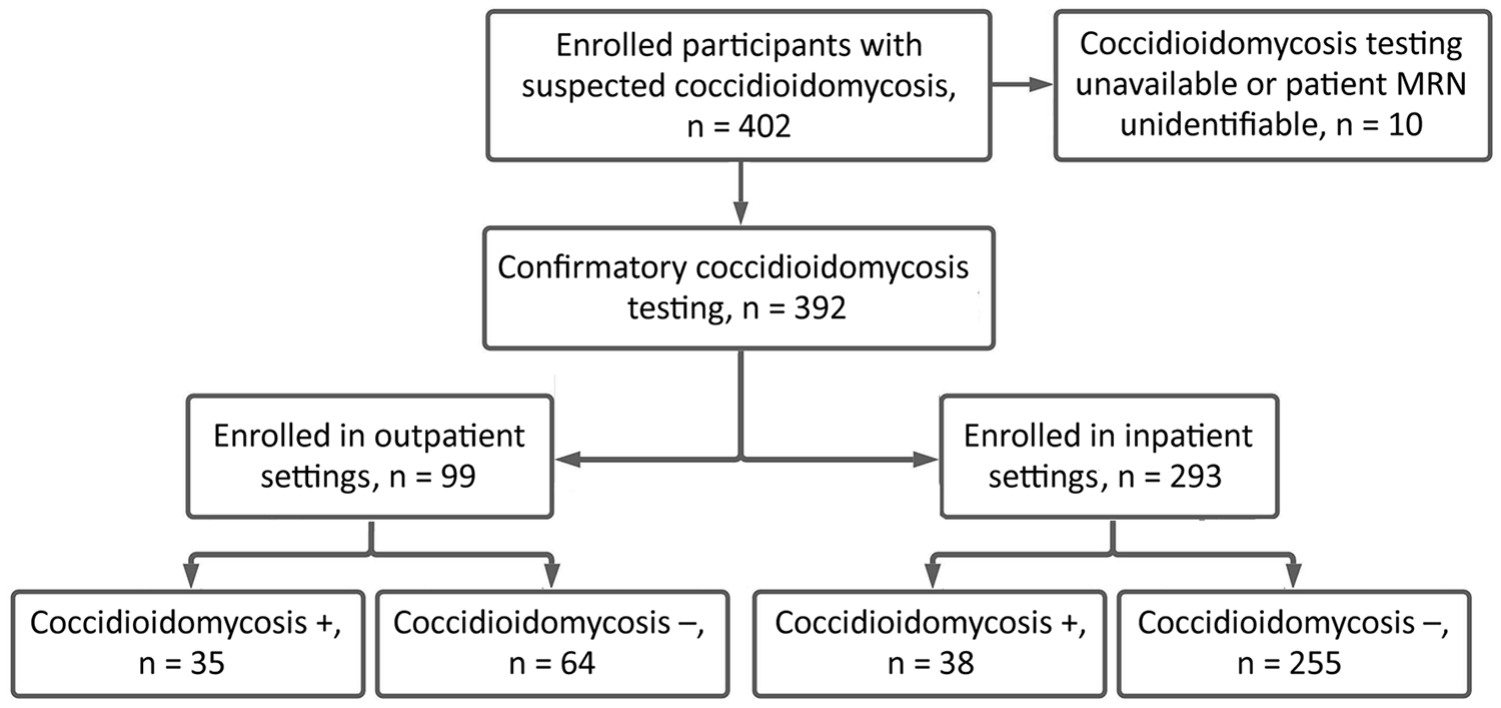
Stratification diagram for suspected coccidioidomycosis among inpatients and outpatients in a cross-sectional study of clinical predictors of coccidioidomycosis, Arizona, USA. Outpatient participants were recruited from emergency departments and affiliated clinics. Inpatient participants were recruited from among hospitalized patients. MRN, medical record number; +, positive; –, negative.

**Table 2:** Characteristics of inpatients and outpatients by confirmed *Coccidioides* diagnosis in a cross-sectional study of clinical predictors of coccidioidomycosis, Arizona, USA*

Characteristics	Outpatient			Inpatient		Total, n = 392	p value	
	Positive, n = 35	Negative, n = 64		Positive, n = 38	Negative, n = 255		Outpatient	Inpatient
Median age, y (range)	57 (24–77)	51 (19–93)		45 (18–83)	58 (18–98)	57 (18–98)	0.534	**0.022**
Sex, no. (%)							0.289	1.000
F	20 (58.8)	29 (46)		18 (47.4)	123 (48.4)	190 (48.8)		
M	14 (41.2)	34 (54)		20 (52.6)	131 (51.6)	199 (51.2)		
Race, no. (%)							0.574	**0.018**
African American	2 (5.9)	4 (6.6)		6 (16.7)	14 (5.6)	26 (6.8)		
AI/AN	4 (11.8)	2 (3.3)		2 (5.6)	9 (3.6)	17 (4.5)		
Asian	1 (2.9)	2 (3.3)		2 (5.6)	2 (0.8)	7 (1.8)		
White	27 (79.4)	52 (85.2)		25 (69.4)	211 (84.7)	315 (82.9)		
Unknown	0	1 (1.6)		1 (2.8)	13 (5.2)	15 (3.9)		
Ethnicity, no. (%)							0.808	1.000
Hispanic	8 (25.8)	18 (30)		10 (27)	69 (27.6)	105 (27.8)		
Non-Hispanic	23 (74.2)	42 (70)		27 (73)	181 (72.4)	273 (72.2)		
Median length of endemic residence, y (range)	10 (0–59)	20 (0–88)		19 (0–78)	22 (0–98)	20 (0–98)	0.091	0.331
Immunocompromised, no. (%)†							0.344	0.076
Y	7 (20.6)	20 (31.2)		17 (44.7)	154 (61.1)	198 (51)		
N	27 (79.4)	44 (68.8)		21 (55.3)	98 (38.9)	190 (49)		
Median length of Illness, d (range)	14 (0–300)	14 (0–5,110)		14 (2–365)	14 (1–8,760)	14 (0–8,760)	0.370	0.972
Symptoms, no. (%)‡								
Fatigue	19 (54.3)	39 (60.9)		27 (71.1)	203 (79.9)	288 (73.7)	0.531	0.209
Cough	22 (62.9)	44 (68.8)		26 (68.4)	164 (64.6)	256 (65.5)	0.656	0.718
Fever	12 (34.3)	24 (37.5)		15 (39.5)	128 (50.4)	179 (45.8)	0.829	0.227
Chest pain	13 (37.1)	26 (40.6)		14 (36.8)	82 (32.3)	135 (34.5)	0.831	0.583
Shortness of breath	13 (37.1)	42 (65.6)		25 (65.8)	172 (67.7)	256 (65.5)	**0.011**	0.853
Headache	9 (25.7)	22 (34.4)		13 (34.2)	116 (45.7)	160 (40.9)	0.497	0.221
Night sweats	15 (42.9)	21 (32.8)		13 (34.2)	104 (40.9)	153 (39.1)	0.384	0.481
Muscle aches	13 (37.1)	28 (43.8)		10 (26.3)	122 (47.8)	163 (41.7)	0.670	**0.014**
Joint pain	13 (37.1)	14 (21.9)		7 (18.4)	82 (32.1)	126 (32.2)	0.156	0.051
Rash	16 (45.7)	3 (4.7)		11 (28.9)	38 (15)	68 (17.3)	**<0.001**	**0.037**
Other	11 (31.4)	27 (42.2)		11 (28.9)	74 (29.1)	123 (31.5)	0.388	1.000
Laboratory tests, median (range)								
Procalcitonin, ng/mL	0.05 (0.012–0.27)	0.10 (0.05–11.59)		0.11 (0.05–92.34)	0.165 (0.02–198.5)	0.11 (0.02–198.5)	**<0.001**	0.617
C-reactive protein, mg/L	7.40 (0.7–260)	17.5 (0.6–266.3)		46.0 (1.4–170.2)	68.0 (0.6–557)	49.00 (0.6–557)	0.090	0.066
ESR, mm/h	15.0 (5–76)	26.0 (1–145)		45.0 (6.0–122.0)	46.0 (4–145)	41.0 (1–145)	0.222	0.427
Leukocytes, × 10^3^ cells/mm^3^	9.8 (4.6–14)	8.9 (3.7–26.5)		9.0 (0.3–24.4)	10.0 (0.1–45.4)	9.90 (0.1–45.4)	0.560	0.481
Hemoglobin, g/dL	13.8 (12.4–15.9)	13.3 (6.9–19.7)		12.0 (7.2–17.4)	12.0 (4.8–18)	12.6 (4.8–19.7)	0.163	0.438
Platelet count, × 10^3^/mm^3^	312.0 (226–457)	238.0 (94–446)		260 (10–520)	239.0 (5–940)	248.0 (5–940)	**<0.001**	0.676
Eosinophil count, × 10^3^/µL	0.39 (0–1.4)	0.1 (0–0.8)		0.2 (0.0–3.0)	0.07 (0.0–4.55)	0.1 (0–4.55)	**<0.001**	**0.015**
Albumin, g/dL	4.05 (2.5–5)	3.9 (1.9–5)		3.0 (1.4–5.0)	3.1 (0.6–6.4)	3.5 (0.6–6.4)	0.483	0.333
Total protein, g/dL	7.3 (6.2–8.7)	7.3 (5.9–9.3)		7.35 (5.4–9.3)	6.95 (2.5–12.0)	7.05 (2.5–12)	0.747	**0.044**

*Inpatient participants were recruited from among hospitalized patients; outpatients were recruited from patients in emergency departments and affiliated clinics. Bold text indicates statistical significance. AI/AN, American Indian/Alaskan Native; ESR, erythrocyte sedimentation rate. †Immunocompromised status was identified as a participant with a weakened immune system at the time of coccidioidomycosis diagnosis, which included participants with type 2 diabetes, HIV/AIDS, lupus, rheumatoid arthritis, or leukemia, and organ transplant recipients and those receiving chemotherapy agents, corticosteroids, and biologic response modifiers. ‡Symptom counts represent the total number of patients reporting the condition.

Among outpatient participants, coccidioidomycosis status did not differ by sex (p = 0.29) or ethnicity (p = 0.81). Length of residence was 10 years for coccidioidomycosis-positive participants and 20 years for coccidioidomycosis-negative participants (p = 0.09) (Table 2). We did not identify differences in length of illness (p = 0.37) or immunocompromised status (p = 0.34). Coccidioidomycosis-positive participants reported shortness of breath significantly less frequently than did coccidioidomycosis-negative participants (p = 0.01), but positive participants reported rash more frequently (p<0.001). Median PCT was significantly lower among positive participants (0.05 ng/mL, range 0.01–0.27 ng/mL) than among -negative participants (0.10 ng/mL, range 0.05–11.6 ng/mL) (p<0.001). Median eosinophil count also was elevated among positive participants (0.39, range 0.0–1.4, interquartile range [IQR] 0.18–0.57) versus negative participants (0.10, range 0.0–0.80, IQR 0.0–0.20) (p<0.001) (Table 2).

Our inpatient population predominantly consisted of coccidioidomycosis-negative participants. Median age was 45 years for coccidioidomycosis-positive and 58 years for coccidioidomycosis-negative inpatient participants (p = 0.02). Coccidioidomycosis diagnosis did not differ for inpatients by sex (p = 1.0) or ethnicity (p = 1.0). Median length of residence was 19 years among positive participants versus 22 years for negative participants (p = 0.33). No difference was identified in median length of illness (p = 0.97). Coccidioidomycosis-positive participants reported muscle aches less frequently than coccidioidomycosis-negative participants (p = 0.01). As we noted in the outpatient population, rash was more frequent among positive participants in the inpatient population (p = 0.04). Median PCT was not statistically lower among positive (0.11 ng/mL, range 0.05–92.3 ng/mL) than negative participants (0.17 ng/mL, range 0.02–198.5 ng/mL) (p = 0.62) (Table 2). We observed lower median CRP levels (46.0 mg/mL, range 1.4–170.2 mg/mL) among positive participants than for negative participants (CRP 68.0 mg/mL, range 0.6–557.0 mg/mL), although the relationship did not meet statistical significance (p = 0.07). Median eosinophil count was elevated (0.2 × 10^3^/µL, range 0.0–3.0 × 10^3^/µL, IQR 0.0–0.30 × 10^3^/µL) among positive participants compared with negative participants (0.07 × 10^3^/µL, range 0.0–4.55 × 10^3^/µL, IQR 0.0–0.20 × 10^3^/µL) (p = 0.015). In contrast to outpatient participants, median total protein was moderately elevated among positive (7.4 g/dL, range 5.4–9.3 g/dL) compared with negative inpatient participants (7.0 g/dL, range 2.5–12.0 g/dL) (p = 0.04) (Table 2).

We developed univariable and multivariable LASSO prediction models for coccidioidomycosis stratified by admission status. Within our outpatient univariable models, positivity was significantly associated with rash (p = 0.006), higher eosinophil count (p = 0.012), and a lower PCT concentration (p = 0.039) (Table 3). Univariate models suggested eosinophilia (>0.50 × 10^3^/µL) is predictive of coccidioidomycosis (Appendix Table 3). Our inpatient univariable models identified higher eosinophil count, higher serum protein, lower age, lower CRP concentration, non-White racial identification, and rash as predictors of coccidioidomycosis, but muscle aches and immunocompromised status were negatively associated with disease (Table 4).

**Table 3:** Characteristics of outpatients in univariable and multivariable models in a cross-sectional study of clinical predictors of coccidioidomycosis, Arizona, USA*

Characteristics	Univariable model			Multivariable model	
	OR (95% CI)	p value		aOR (95% CI)	p value
Symptoms					
Rash	19.64 (2.34–164.67)	**0.006**		9.74 (1.03–92.24)	**0.047**
Shortness of breath	0.43 (0.17–1.09)	0.075		0.36 (0.12–1.07)	0.066
Laboratory tests					
Procalcitonin, ng/mL	0.45 (0.21–0.96)	**0.039**		0.59 (0.25–1.38)	0.222
Platelet count, × 10^3^/mm^3^	1.73 (0.98–3.07)	0.060		1.70 (0.90–3.22)	0.100
Eosinophil count, × 10^3^/µL	2.18 (1.19–4.01)	**0.012**		1.62 (0.79–3.32)	0.186

*Participants were recruited from among patients in emergency departments and affiliated clinics, including 35 coccidioidomycosis-positive and 64 coccidioidomycosis-negative participants. Bold text indicates statistical significance. aOR, adjusted OR; OR, odds ratio.

**Table 4:** Characteristics of inpatients in univariable and multivariable models in a cross-sectional study of clinical predictors of coccidioidomycosis, Arizona, USA*

Characteristics	Univariable model			Multivariable model	
	OR (95% CI)	p value		aOR (95% CI)	p value
Demographics					
Age, y	0.70 (0.50–0.98)	**0.035**		0.72 (0.51–1.03)	0.071
Non-White race	2.42 (1.16–5.04)	**0.018**		2.14 (0.51–1.03)	0.061
Symptoms					
Muscle aches	0.45 (0.22–0.94)	**0.034**		0.38 (0.17–0.84)	**0.017**
Rash	2.29 (1.08–4.84)	**0.030**		2.20 (0.97–4.99)	0.060
Clinical feature					
Immunocompromised	0.49 (0.25–0.94)	**0.033**		0.64 (0.31–1.31)	0.220
Laboratory tests					
C-reactive protein, mg/L	0.66 (0.46–0.94)	**0.023**		0.72 (0.49–1.07)	0.100
Eosinophil count, × 10^3^/µL	1.65 (1.17–2.34)	**0.005**		1.50 (1.02–2.19)	**0.037**
Total protein, g/dL	1.50 (1.08–2.08)	**0.015**		1.30 (0.91–1.87)	0.152

*Participants were recruited from among hospitalized patients, including 38 coccidioidomycosis-positive participants and 255 coccidioidomycosis-negative participants. Bold text indicates statistical significance. Bold text indicates statistical significance. aOR, adjusted odds ratio; OR, odds ratio.

Selected features for our outpatient multivariable model included rash, shortness of breath, PCT, platelet count, and eosinophil count (Table 3); however, only rash was significantly associated with a coccidioidomycosis-positive test result (adjusted OR [aOR] 9.74, 95% CI 1.03–92.24). Outpatient multivariate models did not identify eosinophil count at any level as a predictive marker (Appendix Table 4). 

Our inpatient model identified unique predictive characteristics compared with the outpatient model, including age, race, immunocompromised status, and CRP. Within our inpatient multivariable model, we identified a negative association with self-reported muscle aches (aOR 0.38, 95% CI 0.17–0.84). The model identified elevated eosinophil count as a significant predictor of coccidioidomycosis positivity (aOR 1.50, 95% CI 1.02–2.19; p = 0.037) (Table 4). Eosinophilia was not identified as a significant predictive marker of coccidioidomycosis in our inpatient univariable model, but multivariate models applying lower thresholds indicated eosinophil levels ≥0.20 × 10^3^/µL (200 cells/µL) were predictive of coccidioidomycosis (Appendix Table 4). 

Using cross-validation, our outpatient model yielded an ROC AUC of 78.2% (95% CI 67.2%–89.1%) with a sensitivity of 72.7% and specificity of 69.5%. Our inpatient model yielded an ROC AUC of 64.3% (95% CI 55.2%–72.8%) with a sensitivity of 34.4% and specificity of 87.5% (Table 5).

**Table 5:** Performance metrics for outpatient and inpatient multivariable model in a cross-sectional study of clinical predictors of coccidioidomycosis, Arizona, USA*

Metric	Outpatient	Inpatient
ROC AUC	78.2	64.3
Sensitivity	72.7	34.4
Specificity	69.5	87.5
Positive predictive value	28.6	11.9
Negative predictive value	93.8	96.4
Prevalence	14.4	4.6
Detection rate	10.5	1.6
Detection prevalence	36.6	13.5
Balanced accuracy	71.1	61.0

*ROC AUC, receiver operating characteristic area under the curve.

Features selected in multivariable models were identical in replicated imputation datasets, suggesting consistency in variable selection. Sensitivity analyses performed by removing 198 immunocompetent outpatient participants similarly identified rash and elevated eosinophil count as predictors of coccidioidomycosis positivity (Appendix Table 5). Specificity in our immunocompetent outpatient model was lower (24.0%) than for the full model (69.5%), but sensitivity modestly improved (86.5%) in contrast to the full outpatient model (72.7%) (Appendix Table 6). After removing immunocompromised participants from our inpatient population, we identified no predictive features in either univariable or multivariable models. Univariable models using clinical breakpoints for laboratory measures were directionally consistent with our main results. Outpatient univariable models identified procalcitonin and eosinophil count as major predictors; however, no laboratory predictors were identified in inpatient models after using standardized reference ranges (Appendix Table 3). Multivariable modeling without stratification by admission status identified a similar feature set compared with our inpatient model because of the large sample size of this group relative to our outpatient population (Appendix Table 7). No predictors were identified for either inpatient or outpatient groups using an immunocompromised-only population. Variables identified in models including participants with acute pulmonary symptoms were directionally consistent with our main findings (Appendix Tables 8, 9).

## Discussion

We found preliminary evidence for several markers that could predict coccidioidomycosis based on admission status. Although <40% of outpatient and inpatient participants had rash, our results suggest that rash might support coccidioidomycosis identification better than other symptoms, such as shortness of breath and muscle aches. In outpatient settings, PCT might help differentiate between a bacterial and *Coccidioides* infection. However, for inpatient settings, conventional indicators, including CRP level and immunocompromised status, might be concealed by comorbidities and high inflammatory markers typical to admitted patients and reduce their efficacy as predictive risk factors. Our models suggest elevated eosinophil count could be a viable biomarker to signal coccidioidomycosis in either clinical setting.

Both our univariable analyses and multivariable models among outpatients indicated rash as a major predictor of coccidioidomycosis. Our results were likely driven by the low incidence of rash among coccidioidomycosis-negative participants (4.7%) compared with coccidioidomycosis-positive participants (45.7%). This finding might emphasize the utility of rash as a unique marker of coccidioidomycosis, considering the comparatively low occurrence of this symptom in the outpatient population. Our findings are consistent with previous studies suggesting rash is more frequently identified among coccidioidomycosis cases than among cases of other common respiratory infections (*25*). PCT was negatively associated with positive status, but elevated eosinophil count was a predictive marker of coccidioidomycosis. Laboratory markers were not predictive in our multivariable model; however, low serum PCT levels previously have been reported in persons with coccidioidomycosis (*26*). Lower PCT is consistent with the cell-mediated immune response against *Coccidioides* infection because the production of interferon gamma from type-1 T-helper cells impedes PCT upregulation (*27*). Previous studies also have indicated elevated eosinophil counts among persons with coccidioidomycosis (*28*,*29*). Our results substantiate previous recommendations that eosinophilia heightens suspicion of *Coccidioides* infection (*30*).

Our univariable analyses for inpatients identified negative associations with age, muscle aches, immunocompromised status, and CRP with coccidioidomycosis positivity, but non-White racial status, rash, eosinophil count, and total protein were positive predictive markers of disease. Our multivariable model selected an identical feature set, but only lower incidence of muscle aches and a higher eosinophil count remained statistically significant. Some of our null findings could be explained by the high concentration of immunocompromised participants in the inpatient setting, because these patients often have established coccidioidomycosis risk factors at admission. Furthermore, previous evidence suggests that 20%–50% of specimens from immunocompromised persons test false-negative by *Coccidioides* serologies (*31*); thus, false-negative test results among coccidioidomycosis-negative participants might have been artificially inflated in our study. Of note, older age is a well-established coccidioidomycosis risk factor because of the decline in immune function and higher prevalence of chronic diseases among older persons (*32*); substantial evidence also suggests that immunocompromised persons are more susceptible (*33*). Therefore, the predictive capacity of these risk factors might be limited by the intersecting clinical patterns of coccidioidomycosis and other diseases in the inpatient setting. We also identified CRP as a negative predictor for coccidioidomycosis. As a generalized blood test marker, CRP might have detected higher inflammation for other conditions among inpatients. Like our outpatient results, LASSO selection incorporated eosinophil count into our multivariable model, indicating that eosinophil levels >0.20 × 10^3^/µL might be predictive of *Coccidioides* infection in inpatient settings. Inpatient models did not identify PCT as a negative predictor of coccidioidomycosis.

Our results differ from risk factors previously identified by Yozwiak et al. (*13*), who developed a model using healthy college-aged students. Although these previously identified risk factors might have practical value for estimating relative risk in a healthy population, the inconsistent feature set with our study suggests previous results have limited transferability to a more diverse clinical population. For example, Yozwiak et al. reported male sex, shorter length of residence in coccidioidomycosis-endemic areas, and shorter duration of symptoms as independent risk factors for coccidioidomycosis, which we did not detect as predictors of disease in our study. Yozwiak et al. further reported higher ESR rates and lower lymphocyte levels were associated with disease. Although eosinophil count was indicative of coccidioidomycosis in our study, we did not identify ESR or other cell types as statistically significant predictive markers.

We describe novel coccidioidomycosis prediction models for inpatient and outpatient clinical settings using an agnostic feature selection technique. We constructed models by using data from our previous cross-sectional study and leveraged these data to substantiate risk factors previously associated with coccidioidomycosis, including clinical, demographic, and laboratory variables. We identified markers that might identify coccidioidomycosis before diagnostic testing and distinctive predictive features based on admission status. We stratified models by inpatient and outpatient groups because of the unique features identified within each clinical setting. Our study identified several clinical features in outpatient and inpatient settings, but screening for *Coccidioides* in endemic settings remains invaluable. Although negative clinical features, such as PCT, muscle aches, or shortness of breath, might be indicative of an alternative diagnosis, we emphasize that the presence of these markers should not deter testing.

Limitations of our study include a reduced sample size used to develop our models, in part due to our stratification, which might have hampered our ability to accurately estimate predictive markers of coccidioidomycosis. We were further unable to apply clinical breakpoints for laboratory measures because of reduced granularity of binary measures and therefore report the effect of continuous variables. We attempted to minimize feature selection biases by using LASSO to construct models; LASSO offers several benefits over alternative feature selection methods, but our impartial approach might have inappropriately eliminated collinear or other necessary control variables. We additionally recognize that participants with established coccidioidomycosis markers might have been preferentially tested during enrollment, resulting in selection bias, and influencing marker selection. The relative infrequency of identified features further hinders the clinical utility of leveraging these markers to identify coccidioidomycosis and emphasizes the importance of diagnostic testing.

Our study’s strengths include that we used a novel multidimensional dataset to evaluate established and suspected coccidioidomycosis risk factors. Stratification reveals substructures within clinical settings that could improve disease identification and diagnosis. Our sensitivity analyses using immunocompetent patients further increases confidence in selected features, because rash and higher eosinophil count were similarly predictive of coccidioidomycosis in the outpatient setting.

Public health recommendations are to test for *Coccidioides* among patients with pneumonialike symptoms in endemic areas. However, the complex and often nonspecific clinical manifestations of coccidioidomycosis indicate a need to improve disease identification. Coupled with the introduction of coronavirus disease in 2019, differentiating between coccidioidomycosis and other pneumonias remains vital for the rapid diagnosis and treatment of disease. The limited accuracy of our models, however, indicate the need for a more robust data source for model development. Replication in a larger clinical study incorporating other endemic regions could provide insight into additional predictive markers for more specific clinical manifestations. Our study identifies surrogate markers in a clinical setting that might provide a developmental framework for future predictive models. 

In conclusion, we developed prediction models for multiple clinical settings to support identification of coccidioidomycosis before diagnostic testing. Prediction models could guide the clinical decision-making process to test for coccidioidomycosis, expedite identification of more serious disease complications, and decrease the use of unnecessary diagnostic tests or antimicrobial agents.

Appendix Additional information on a cross-sectional study of clinical predictors of coccidioidomycosis, Arizona, USA
