# Supplementary material for: Cross-Sectional Study of Clinical Predictors of Coccidioidomycosis, Arizona, USA
Source: Emerg Infect Dis. 2022 Jun;28(6):1091–100. doi: 10.3201/eid2806.212311 (PMC9155888; doi:10.3201/eid2806.212311)
Supplement: Appendix — Additional information on a cross-sectional study of clinical predictors of coccidioidomycosis, Arizona, USA [file 21-2311-Techapp-s1.pdf]

Article DOI: https://doi.org/10.3201/eid2806.212311

Cross-Sectional Study of Clinical Predictors of Coccidioidomycosis, Arizona, USA

Appendix

Appendix Table 1. Missing observations and imputation methods for model features in a cross-sectional study of clinical predictors of coccidioidomycosis, Arizona, USA*

Characteristics	Missing observations, no. (%)	Imputation method
Demographics		
Age	4 (1.0)	Predictive mean matching
Sex	2 (0.5)	Logistic regression
Race (non-White)	11 (2.8)	Polytomous regression
Ethnicity	13 (3.3)	Logistic regression
Length of endemic residence	15 (3.8)	Predictive mean matching
Clinical Features		
Electronic medical record location	0	NA
Admission Status	0	NA
Immunocompromised	3 (0.8)	Logistic regression
Duration of illness	3 (0.8)	Predictive mean matching
Symptoms		
Fatigue	0	NA
Cough	0	NA
Fever	0	NA
Chest pain	0	NA
Shortness of breath	0	NA
Headache	0	NA
Night sweats	0	NA
Muscle Aches	0	NA
Joint pain	0	NA
Rash	0	NA
Other	0	NA
Lab Measures		
Procalcitonin	13 (3.3)	Predictive mean matching
C-reactive protein	18 (4.6)	Predictive mean matching
Erythrocyte sedimentation rate	14 (3.6)	Predictive mean matching
Leukocyte count	22 (5.6)	Predictive mean matching
Hemoglobin	22 (5.6)	Predictive mean matching
Platelet count	22 (5.6)	Predictive mean matching
Eosinophil count	63 (16.1)	Predictive mean matching
Albumin	53 (13.6)	Predictive mean matching
Total protein	53 (13.6)	Predictive mean matching
Outcome		
Coccidioidomycosis	0	NA

*NA, not applicable.

Appendix Table 2. Patient symptoms and laboratory measures for confirmed cases in a cross-sectional study of clinical predictors of coccidioidomycosis, Arizona, USA*

Characteristics	CM-positive, n = 73	CM-negative, n = 319	Total, n = 392	p value
Median length of Illness, d (range)	14 (0–365)	14 (0–8,760)	14 (0–8,760)	0.676
Symptoms, no. (%)†				
Fatigue	46 (63)	242 (76.1)	288 (73.7)	**0.027**
Cough	48 (65.8)	208 (65.4)	256 (65.5)	1.000
Fever	27 (37)	152 (47.8)	179 (45.8)	0.118
Chest pain	27 (37)	108 (34)	135 (34.5)	0.684
Shortness of Breath	42 (57.5)	214 (67.3)	256 (65.5)	**0.021**
Headache	22 (30.1)	138 (43.4)	160 (40.9)	0.047
Night sweats	28 (38.4)	125 (39.3)	153 (39.1)	1.000
Muscle aches	23 (31.5)	140 (44)	163 (41.7)	0.065
Joint pain	20 (27.4)	106 (33.3)	126 (32.2)	0.405
Rash	27 (36.9)	41 (12.9)	68 (17.3)	**<0.001**
Other	22 (30.1)	101 (31.8)	123 (31.5)	0.889
Laboratory Tests, median (range)				
Procalcitonin, ng/mL	0.10 (0.05–92.34)	0.17 (0.02–198.5)	0.11 (0.02–198.5)	**<0.001**
C-reactive protein, mg/L	28.90 (0.7–260)	55.30 (0.6–557)	49.00 (0.6–557)	**<0.001**
Erythrocyte sedimentation rate, mm/hr	29.00 (5–122)	43.00 (1–145)	41.00 (1–145)	**0.003**
Leukocyte count, K/mm^3	9.75 (0.3–24.4)	10.10 (0.1–45.4)	9.90 (0.1–45.4)	0.466
Hemoglobin, g/dL	13.50 (7.2–17.4)	12.40 (4.8–19.7)	12.60 (4.8–19.7)	**0.008**
Platelet count, K/mm^3	268.50 (10–520)	238.00 (5–940)	248.00 (5–940)	**0.010**
Eosinophil count, K/µL	0.20 (0.0–3)	0.10 (0–4.55)	0.10 (0–4.55)	**<0.001**
Albumin, g/dL	3.50 (1.4–5)	3.50 (0.6–6.4)	3.50 (0.6–6.4)	0.211
Total protein, g/dL	7.30 (5.4–9.3)	7.00 (2.5–12)	7.05 (2.5–12)	**0.040**

*CM, Coccidioidomycosis.
†Symptom counts represent the total number of patients reporting the condition.

Appendix Table 3. Univariable models using clinical thresholds in outpatient and inpatient settings in a cross-sectional study of clinical predictors of coccidioidomycosis, Arizona, USA*

		Outpatient		Inpatient	
Laboratory Tests	Reference range	OR (95% CI)	p value	OR (95% CI)	p value
Procalcitonin, ng/mL	<0.50	0.07 (0.01–0.55)	**0.011**	0.83 (0.37–1.85)	0.651
C-reactive protein, mg/L	2.9–8.0	0.48 (0.21–1.12)	0.091	0.82 (0.30–2.27)	0.699
Erythrocyte sedimentation rate, mm/hr	male <50: <16.0<br>male ≥50: <21.0<br>female <50: <21.0<br>female ≥50: <31.0	0.56 (0.24–1.30)	0.177	0.54 (0.26–1.13)	0.103
Leukocyte count, K/mm^3	4.5–11.0	1.11 (0.44–2.77)	0.830	0.69 (0.34–1.40)	0.309
Hemoglobin, g/dL	male: 13.8–17.2<br>female: 12.1–15.1	0.91 (0.21–3.87)	0.894	2.09 (0.55–7.97)	0.280
Platelet count, K/mm^3	150.0–400.0	1.20 (0.42–3.45)	0.729	1.63 (0.80–3.33)	0.182
Eosinophil count, K/µL	<0.50	6.02 (1.48–24.48)	**0.012**	2.81 (0.94–8.39)	0.064
Albumin, g/dL	3.4–5.4	1.68 (0.52–5.47)	0.386	1.56 (0.77–3.15)	0.216
Total protein, g/dL	6.0–8.3	1.25 (0.33–4.75)	0.746	2.60 (0.88–7.68)	0.084

*Outpatient participants were recruited from among patients in emergency departments and affiliated clinics, including 24 coccidioidomycosis-positive and 37 coccidioidomycosis-negative participants. Inpatient participants were recruited from among hospitalized patients, including 38 coccidioidomycosis-positive and 255 coccidioidomycosis-negative participants Bold text indicates statistical significance. OR, odds ratio.

Appendix Table 4. Univariable models for eosinophil count clinical thresholds in outpatient and inpatient settings in a cross-sectional study of clinical predictors of coccidioidomycosis, Arizona, USA*

Admission status	Eosinophil count, K/µL	No. cases	No. controls	OR (95% CI)	p value	AOR (95% CI)	p value
Outpatient	<0.50	11	88	6.02 (1.48–24.48)	**0.012**	1.48 (0.31–6.93)	0.621
	<0.45	16	83	5.41 (1.70–17.23)	**0.004**	2.52 (0.57–11.11)	0.222
	<0.40	19	80	5.71 (1.93–16.90)	**0.002**	2.32 (0.59–9.07)	0.227
	<0.35	21	78	5.43 (1.93–15.30)	**0.001**	2.24 (0.58–8.61)	0.239
	<0.30	29	70	5.10 (2.02–12.90)	**0.001**	2.35 (0.71–7.79)	0.162
	<0.25	30	69	5.72 (2.26–14.49)	**<0.001**	2.72 (0.84–8.78)	0.095
	<0.20	45	54	3.66 (1.54–8.72)	**0.003**	1.86 (0.63–5.55)	0.263
	<0.15	48	51	4.46 (1.82–10.89)	**0.001**	2.48 (0.83–7.39)	0.102
	<0.10	65	34	2.92 (1.11–7.66)	**0.030**	1.91 (0.58–6.28)	0.286
Inpatient	<0.50	18	274	2.81 (0.94–8.39)	0.064	2.21 (0.72–6.81)	0.166
	<0.45	24	268	2.46 (0.91–6.65)	0.076	2.08 (0.69–6.32)	0.194
	<0.40	36	256	2.15 (0.90–5.15)	0.085	1.62 (0.62–4.23)	0.328
	<0.35	36	256	2.15 (0.90–5.15)	0.085	1.62 (0.62–4.23)	0.328
	<0.30	58	234	2.42 (1.15–5.08)	**0.020**	2.24 (0.98–5.10)	0.054
	<0.25	58	234	2.42 (1.15–5.08)	**0.020**	2.24 (0.98–5.10)	0.054
	<0.20	89	203	2.63 (1.31–5.26)	**0.006**	2.41 (1.12–5.17)	**0.024**
	<0.15	93	99	2.43 (1.22–4.85)	**0.012**	2.21 (1.03–4.73)	**0.041**
	<0.10	143	149	1.94 (0.96–3.93)	0.064	1.83 (0.85–3.95)	0.124

*Outpatient participants were recruited from among patients in emergency departments and affiliated clinics, including 35 coccidioidomycosis-positive and 64 coccidioidomycosis-negative participants. Inpatient participants were recruited from among hospitalized patients, including 38 coccidioidomycosis-positive and 255 coccidioidomycosis-negative participants Bold text indicates statistical significance. AOR, adjusted odds ratio; OR, odds ratio.

Appendix Table 5. Multivariable models for immunocompetent participants in outpatient settings in a cross-sectional study of clinical predictors of coccidioidomycosis, Arizona, USA*

	Univariable		Multivariable	
Characteristics	OR (95% CI)	p value	AOR (95% CI)	p value
Symptoms				
Rash	11.37 (1.27–101.45)	**0.029**	7.47 (0.76–72.91)	0.084
Laboratory Tests				
Eosinophil count, K/µL	2.31 (1.17–4.59)	**0.016**	1.97 (0.94–4.14)	0.073

*Outpatient participants were recruited from among patients in emergency departments and affiliated clinics, including 24 coccidioidomycosis-positive and 37 coccidioidomycosis-negative participants. Bold text indicates statistical significance. AOR, adjusted odds ratio; OR, odds ratio.

Appendix Table 6. Performance metrics of immunocompetent outpatient multivariable model in a cross-sectional study of clinical predictors of coccidioidomycosis, Arizona, USA*

Metric	Immunocompetent outpatient
ROC AUC	62.4
Sensitivity	86.5
Specificity	24.0
Positive predictive value	62.8
Negative predictive value	54.6
Prevalence	59.7
Detection rate	51.6
Detection prevalence	82.3
Balanced accuracy	55.2

*ROC AUC, receiver operating characteristic area under the curve.

Appendix Table 7. Multivariable model by any admission status models in a cross-sectional study of clinical predictors of coccidioidomycosis, Arizona, USA*

Characteristics	AOR (95% CI)	p value
Age	0.77 (0.56–1.05)	0.097
Length of residence, y	0.83 (0.59–1.17)	0.286
Admission status, outpatient	3.37 (1.58–7.19)	**0.002**
Immunocompromised status	0.59 (0.30–1.16)	0.129
Clinical features		
Shortness of breath	0.63 (0.34–1.17)	0.146
Headache	0.59 (0.30–1.16)	0.126
Muscle aches	0.56 (0.29–1.07)	0.080
Rash	2.67 (1.27–5.64)	**0.010**
Laboratory tests		
C-reactive protein, mg/L	0.68 (0.48–0.97)	**0.033**
Leukocyte count, K/mm^3	0.77 (0.54–1.11)	0.163
Hemoglobin, g/dL	1.27 (0.88–1.83)	0.204
Eosinophil count, K/µL	1.61 (1.14–2.26)	**0.006**
Total protein, g/dL	1.75 (1.21–2.54)	**0.003**
Albumin, g/dL	0.53 (0.34–0.82)	**0.005**

*Participants were recruited from among patients in hospitals, emergency departments, and affiliated clinics, including 73 coccidioidomycosis-positive and 319 coccidioidomycosis-negative participants. Bold text indicates statistical significance. AOR, adjusted odds ratio.

Appendix Table 8. Univariable and multivariable models in outpatient settings among participants with acute primary pulmonary disease in a cross-sectional study of clinical predictors of coccidioidomycosis, Arizona, USA*

	Univariable		Multivariable	
Characteristics	OR (95% CI)	p value	AOR (95% CI)	p value
Demographics				
Non-White race	3.14 (1.00–9.92)	0.051	12.97 (1.44–116.87)	**0.022**
Symptoms				
Rash	25.00 (4.84–129.13)	**<0.001**	75.55 (3.82–1,494.09)	**0.005**
Shortness of breath	0.21 (0.07–0.61)	**0.004**	0.13 (0.02–0.78)	**0.026**
Clinical Features				
Immunocompromised	0.21 (0.04–0.98)	**0.048**	0.13 (0.02–1.06)	0.056
Laboratory Tests				
Platelet count, K/mm^3	2.77 (1.36–5.64)	**0.005**	1.91 (0.57–6.39)	0.296
Eosinophil count, K/µL	2.18 (1.19–4.01)	**0.012**	4.73 (1.03–21.84)	**0.046**

*Outpatient participants were recruited from among patients in emergency departments and affiliated clinics, including 22 coccidioidomycosis-positive and 55 coccidioidomycosis-negative participants. Bold text indicates statistical significance. AOR, adjusted odds ratio; OR, odds ratio.

Appendix Table 9. Univariable and multivariable models in inpatient settings among participants with acute primary pulmonary disease in a cross-sectional study of clinical predictors of coccidioidomycosis, Arizona, USA*

	Univariable		Multivariable	
Characteristics	OR (95% CI)	p value	AOR (95% CI)	p value
Demographics				
Age, y	0.59 (0.38–0.92)	**0.020**	0.58 (0.37–0.90)	**0.016**
Symptoms				
Rash	25.00 (4.84–129.13)	**<0.001**	4.07 (1.37–12.06)	**0.011**
Muscle aches	0.21 (0.07–0.63)	**0.005**	0.18 (0.05–0.59)	**0.005**
Night sweats	0.31 (0.11–0.86)	**0.024**	0.33 (0.11–1.02)	0.054

*Inpatient participants were recruited from among hospitalized patients, including 25 coccidioidomycosis-positive and 190 coccidioidomycosis-negative participants Bold text indicates statistical significance. AOR, adjusted odds ratio; OR, odds ratio.

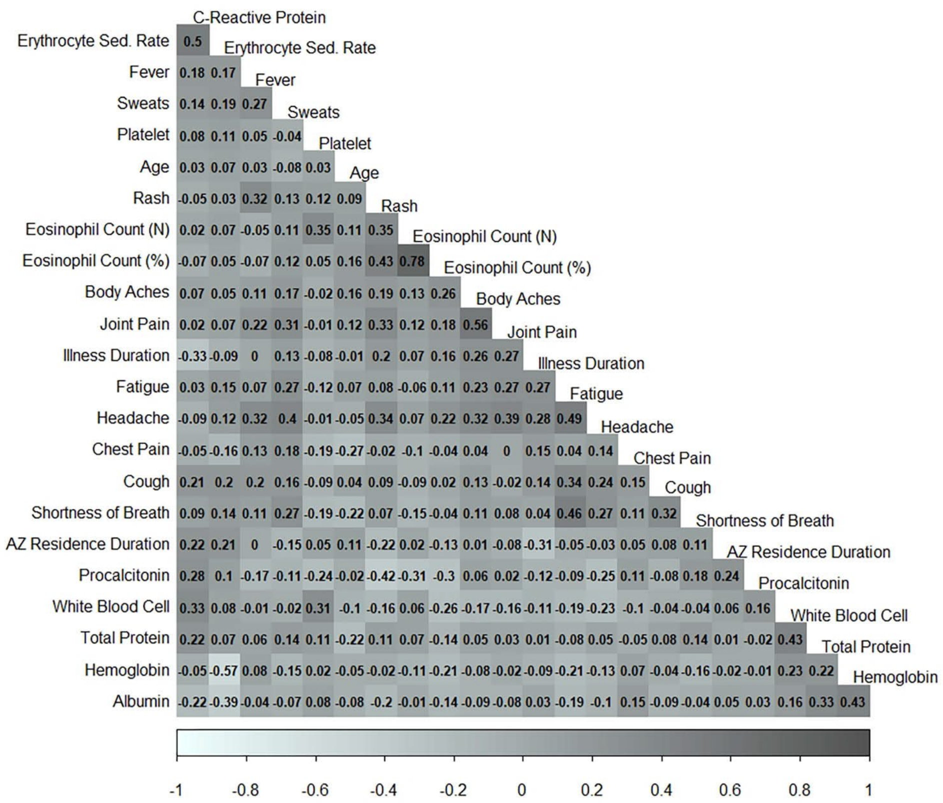

Appendix Figure 1. Collinearity matrix of outpatient participants in a cross-sectional study of clinical predictors of coccidioidomycosis, Arizona, USA. Scale bar indicates the magnitude of pair-wise correlation between variables. AZ, Arizona (USA); Sed., sedimentation.

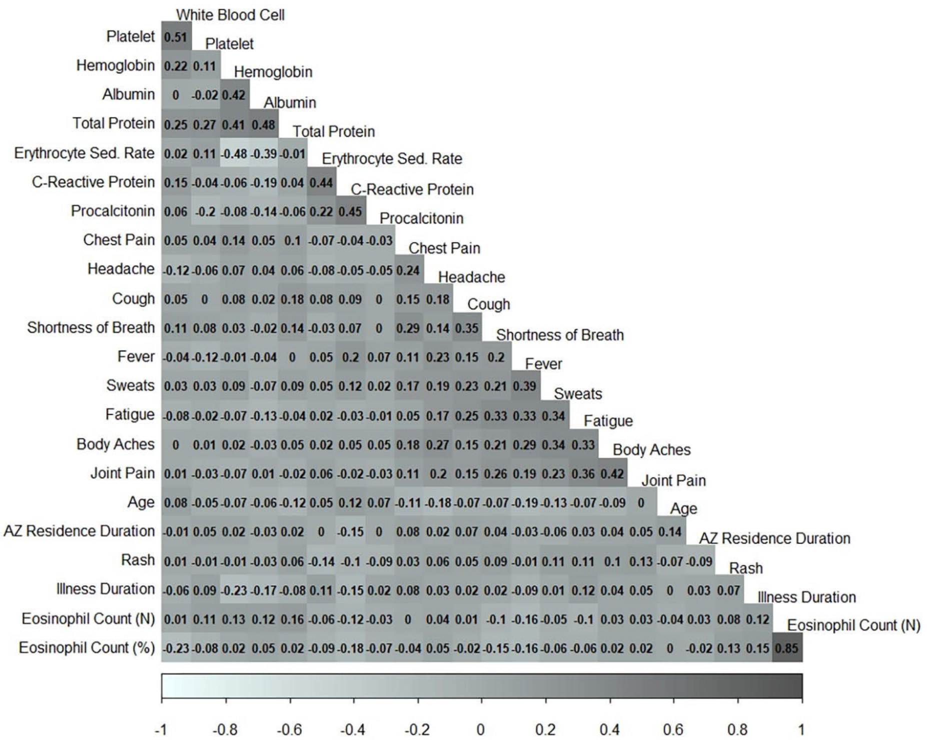

Appendix Figure 2. Collinearity matrix of inpatient participants in a cross-sectional study of clinical predictors of coccidioidomycosis, Arizona, USA. Scale bar indicates the magnitude of pair-wise correlation between variables. AZ, Arizona (USA); Sed., sedimentation.
